# Interval-Level Validation of a Wearable Dry-Electrode ECG Biosensor Platform: A Proof-of-Concept Study

**DOI:** 10.3390/bios16090481

**Published:** 2026-09-01

**Authors:** Avshalom Shaffer, Daniel Possti, Yizhaq Shmayahu, Deganit Barak-Shinar, Roy Beigel, David Hochstein, Rona Haker, Yael Hanein, Hila Meiri, Oliana Vazhgovsky, Shai Tejman-Yarden

**Affiliations:** 1 The Engineering Medical Research Lab, Sheba Medical Center, Ramat Gan 52621, Israel; 2 The Edmond J. Safra International Congenital Heart Center, Sheba Medical Center, Ramat Gan 52621, Israel; 3 The Dina Recanati School of Medicine, Reichman University, Herzliya 4610101, Israel; 4 X-Trodes Ltd., Herzliya 4673304, Israelyaelha@tauex.tau.ac.il (Y.H.); 5 The Leviev Heart Institute, Sheba Medical Center, Ramat Gan 52621, Israel; 6 Internal Medicine Department C, Sheba Medical Center, Ramat Gan 52621, Israel; 7 School of Electrical and Computer Engineering, Tel Aviv University, Tel Aviv 6997801, Israel; 8 Department of General Surgery and Transplantation, Sheba Medical Center, Ramat Gan 52621, Israel

**Keywords:** wearable ECG, dry electrodes, biosensors, cardiac monitoring, RR interval, P-to-R peak interval, signal validation, Bland–Altman analysis

## Abstract

Wearable dry-electrode electrocardiographic (ECG) systems may enable longer and more comfortable rhythm monitoring, but their interval-level measurement fidelity requires validation against reference acquisition systems. In this single-center proof-of-concept study, 20 participants (10 healthy volunteers and 10 cardiology patients) underwent simultaneous ECG recordings with a U.S. Food and Drug Administration (FDA)-cleared wearable dry-electrode platform (X-trodes System M) and a wired reference system (MP150 with ECG100C module, Biopac Systems). Three-minute paired segments were recorded and underwent temporal synchronization, bandpass filtering, baseline correction, automated discrete-wavelet peak detection in NeuroKit2 (version 0.2.10), and standardized manual adjudication. After predefined exclusions for temporal alignment and P-wave detection quality, agreement was assessed in nine cardiac and ten healthy participants for RR intervals and in seven cardiac and eight healthy participants for P-to-R peak intervals. RR interval agreement was excellent in the healthy and cardiac cohorts, with a mean bias of 0.4 ± 3.1 ms and 0.1 ± 4.6 ms and mean absolute error (MAE) of 0.97 ms and 1.28 ms, respectively. Intraclass correlation coefficient (ICC) was over 0.99 for both cohorts. P-to-R peak interval agreement was strong, with an MAE of 1.76 ms and 7.14 ms and ICC of 0.96 and 0.98, respectively. These findings provide preliminary evidence of interval-level agreement for the X-trodes wearable dry-electrode ECG biosensor platform under controlled, short-duration daytime conditions. Because the matched interval pairs were clustered within a small number of participants, the findings should be regarded as proof-of-concept evidence supporting larger ambulatory validation studies rather than as definitive clinical validation.

## 1. Introduction

Continuous ambulatory electrocardiographic (ECG) monitoring is a cornerstone of contemporary cardiovascular care. It enables early arrhythmia detection, risk stratification, and longitudinal rhythm surveillance that cannot be achieved with brief clinic-based recordings [1,2]. Guidelines from major cardiovascular societies endorse prolonged rhythm monitoring for the management of atrial fibrillation, atrioventricular conduction delay, and related conditions [3,4,5].

Wearable ECG platforms based on dry-contact epidermal electrodes make such prolonged recordings practical, permitting longer ambulatory monitoring with less patient burden than conventional gel-based systems [6,7,8,9]. Unlike gel electrodes, which are susceptible to impedance drift as the conductive gel dehydrates, dry-electrode systems are designed for long-term skin conformity and impedance stability and cause less skin irritation during extended wear [10,11]. Published validation studies have focused mainly on arrhythmia detection, with beat-by-beat interval-level agreement against a simultaneous reference system reported comparatively less often [6,7,8]; patients with conduction abnormalities or structural heart disease, for whom interval accuracy has direct clinical implications, are also less frequently represented. Interval-level fidelity, however, is a prerequisite for HRV-based risk stratification, monitoring of atrioventricular conduction delay, and rhythm surveillance in electrophysiologically complex patients [12].

Selecting an electrode technology for continuous, wearable ECG requires balancing four demands: a skin–electrode contact impedance that is both low and stable over time; resistance to motion artifacts; comfort during prolonged wear; and reliability across multi-hour to multi-day recordings. A recent ECG-specific review of dry-electrode types provides an overarching comparison [13]. Rigid dry-metal electrodes (stainless steel, silver, and platinum) are durable and reusable and present a comparatively low impedance, lower than porous polymer or fabric electrodes, but any electrode movement markedly degrades the signal because they cannot conform to the skin [14,15]. Conductive-polymer and elastomer electrodes (carbon-loaded PDMS/silicone, PEDOT:PSS) are flexible and more comfortable, with a moderate, conformity-dependent impedance, but their long-term use is limited by mechanical durability and possible skin irritation [14]. Textile electrodes that can be knitted, woven, or embroidered from silver-coated or stainless-steel yarns are the most comfortable and least obtrusive dry option. They can be integrated into launderable garments and have been applied clinically for ambulatory rhythm monitoring after catheter ablation [16,17]. Because the yarns contact the skin only at discrete fibre points, their skin–electrode impedance is comparatively high. Moreover, it depends on electrode area, the contact pressure and positioning maintained by the garment and skin moisture [18,19]. Consequently, contact quality varies between wearers and drifts within a recording [16]. Contact is sustained by garment tension rather than adhesion, leaving the interface vulnerable to relative motion between the yarns and the skin—the dominant source of signal degradation in ambulatory textile recordings, more so than the elevated impedance itself [15,16]. Microneedle arrays that bypass the stratum corneum achieve very low impedance and are relatively insensitive to motion, but their skin-penetrating design adds fabrication complexity and requires validation of long-term skin safety [20,21]. Capacitive, non-contact electrodes can record through clothing without direct skin contact, but require high-input-impedance amplifiers and are highly susceptible to motion artifacts and baseline drift, limiting ambulatory reliability [14,22,23]. Soft screen-printed epidermal arrays occupy a favorable middle ground: printed on thin, skin-conformal adhesive substrates, they provide a low-to-moderate, stable impedance without conductive gel or skin abrasion and move with the skin, minimizing motion artifacts, at the cost of a single-use adhesive [9,24,25]. This combination of conformity, gel-free application, comfort, and stability under movement gives the screen-printed epidermal approach characteristics relevant to prolonged wearable ECG applications. The platform by X-trodes, originally developed and validated in the Neuro-engineering Laboratory at the Center for Nanoscience and Nanotechnology, Tel Aviv University, and subsequently cleared by the FDA for surface electrophysiology, was therefore selected for evaluation in this study.

**Figure 1:**
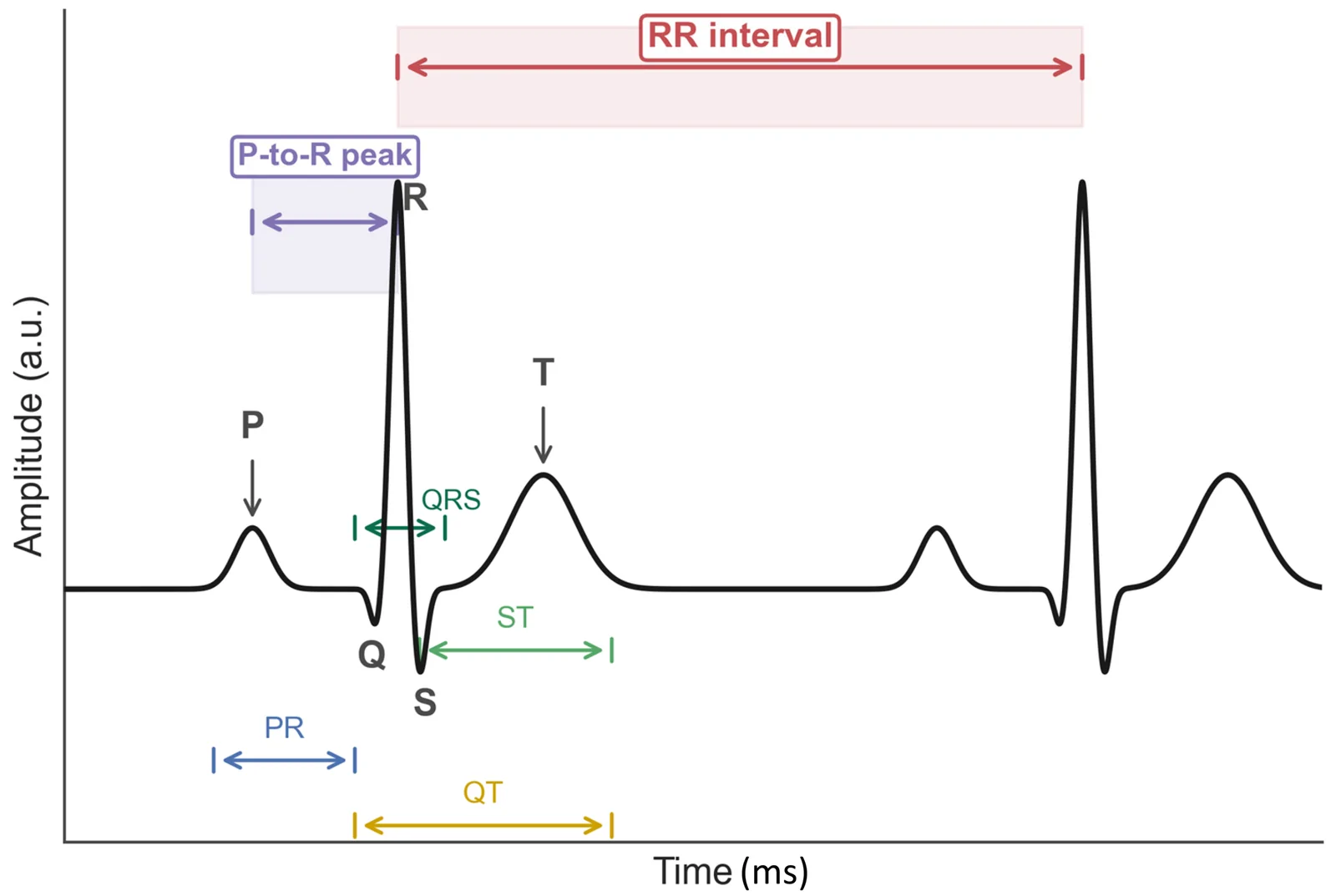
Schematic of an ECG waveform shows the principal deflections (P wave, QRS complex, and T wave) and the intervals derived from them. The RR interval and the P-to-R peak interval, the two parameters analyzed in this study, are highlighted.

Establishing whether such a platform can measure clinical intervals accurately requires defining the intervals themselves. Each cardiac cycle produces three principal deflections on the ECG: the P wave (atrial depolarization), the QRS complex (ventricular depolarization), and the T wave (ventricular repolarization) (Figure 1). The RR interval, one of the most important parameters derived from the ECG, underpins arrhythmia detection and heart-rate-variability (HRV) analysis [26]. The P-to-R peak interval is an algorithmically reproducible timing metric defined between the P-wave peak and the following R peak. It is distinct from the conventional diagnostic PR interval measured from P-wave onset to QRS onset [27] and was selected here as a single-channel fiducial-point metric. Accurate measurement of both intervals is nonetheless difficult in patients with structural heart disease or conduction abnormalities: P-wave timing is sensitive to P-wave amplitude and morphology, particularly when P waves are of low amplitude or partially obscured, and RR measurement can be affected by QRS morphology changes during aberrant conduction or ventricular arrhythmia [27]. These are precisely the conditions under which a wearable platform’s interval fidelity must be demonstrated. Recent reviews confirm that wearable dry-electrode and epidermal ECG is now an established and rapidly maturing field, and consistently identify skin–electrode impedance, contact stability, motion, and perspiration as the principal determinants of real-world signal quality [28,29,30]; low-motion-artifact epidermal recording and multi-day monitoring in cardiac patients have also been demonstrated [31,32].

To address this gap, this proof-of-concept study compared RR and P-to-R peak interval measurements obtained from simultaneous recordings of the X-trodes wearable dry-electrode platform and a wired reference ECG system, in healthy volunteers and in cardiology patients with structural heart disease and/or conduction abnormalities. The study was designed to assess interval-level measurement fidelity and was not intended to evaluate arrhythmia classification, ischemia detection, or clinical diagnostic outcomes. Wearable dry-electrode ECG technology is not itself novel; its specific contribution here is the simultaneous, beat-matched assessment of interval-level agreement between a screen-printed, skin-conformal dry-electrode platform and a wired reference system in healthy participants and in patients with structural heart disease or conduction abnormalities, evaluated with Bland–Altman analysis, mean absolute error (MAE), the intraclass correlation coefficient (ICC), and Pearson correlation (r) rather than correlation alone.

## 2. Materials and Methods

### 2.1. Study Design and Participants

This paired-comparison, proof-of-concept study was conducted at Sheba Medical Center, Ramat Gan, Israel. Twenty participants were enrolled: 10 healthy adult volunteers with no known cardiac history and 10 consecutive patients referred to the cardiology outpatient clinic or admitted to the cardiology ward were recruited. All participants provided their written informed consent. Enrollment was intentionally inclusive of individuals with structural heart disease and conduction abnormalities to evaluate device performance across a clinically representative and electrophysiologically diverse population. All recordings were obtained during routine clinical evaluations or hospitalizations and did not influence patient management. This study was approved by the Sheba Medical Center Institutional Review Board (IRB 9105-22-SMC; approval date: March 2025). This was a paired-comparison proof-of-concept study of interval-level measurement agreement; participants were not assigned to therapeutic interventions, and the study was not intended to evaluate arrhythmia classification, diagnostic accuracy, or clinical outcomes.

### 2.2. ECG Acquisition Systems

**Figure 2:**
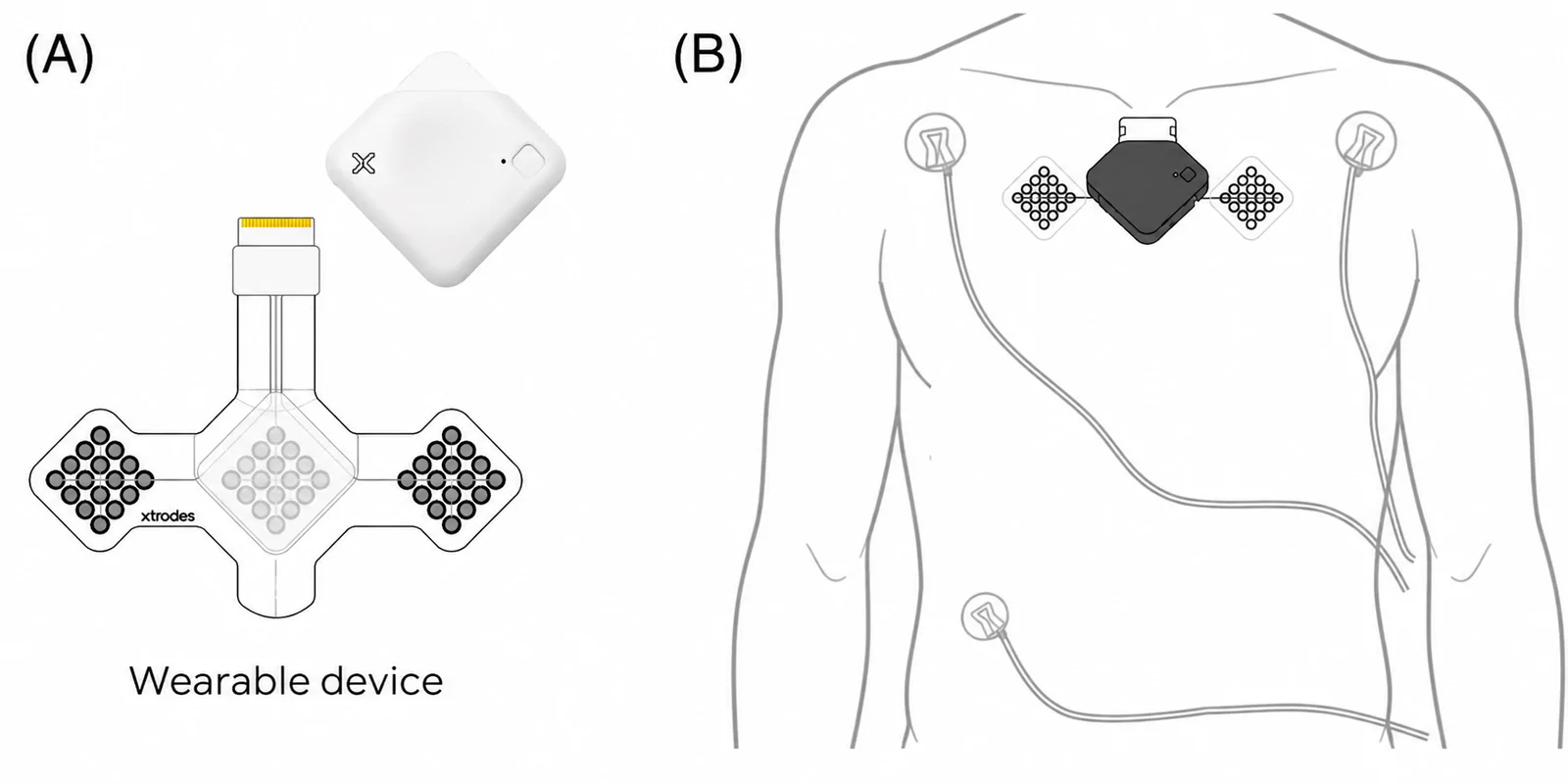
System overview. (**A**) Components of the X-trodes wearable ECG system: the flexible electrode array and the wireless data acquisition unit (DAU). (**B**) Schematic of the experimental setup illustrating concurrent placement of the wearable patch on the anterior chest and the wired reference system (Biopac) Ag/AgCl electrodes.

Simultaneous daytime ECG recordings were obtained using: (1) a U.S. Food and Drug Administration (FDA)-cleared wearable dry-electrode platform, X-trodes System M (X-trodes Ltd., Herzliya, Israel) [33], and (2) a wired reference system, the MP150 data acquisition unit with an ECG100C amplifier module (Biopac Systems, Goleta, CA, USA). Both systems recorded concurrently to ensure beat-to-beat temporal alignment and minimize within-session physiological variability, as illustrated in Figure 2. All recordings were conducted during the daytime hours while the participants were awake and resting. Paired 3-min segments were selected for analysis. This controlled, short-duration acquisition protocol was designed for proof-of-concept validation, not an extended ambulatory or nocturnal monitoring scenario.

### 2.3. The Wearable System

The X-trodes electrode array has been described in detail elsewhere [24,25]. Briefly, carbon electrodes and silver interconnects are screen-printed on a thin, flexible substrate film, forming the wearable array that connects to a data acquisition unit (Figure 2A).

A double-sided adhesive film adheres the array to the skin and electrically insulates the silver traces. Each array incorporates two ECG electrodes positioned on the anterior chest wall, one at the left parasternal border and one at the mid-clavicular line of the left hemithorax. The inter-electrode separation was approximately 10 cm, forming a single differential bipolar recording channel that approximates a modified precordial lead (Figure 2B). The carbon-based dry-electrode design avoids a gel interface, which may be susceptible to dehydration-induced impedance drift during extended recordings [11]. A right-leg drive (RLD) electrode provides common-mode noise rejection.

A miniature proprietary data acquisition unit (DAU; 48 × 38 × 19 mm; 28 g) is connected directly to the electrode array. The DAU records bipolar ECG with a noise floor of 0.79 μV root-mean-square (RMS) (0 to 1048 Hz) and 0.23 μV RMS (0.05 to 70 Hz), a sampling rate of 4000 samples/s, 24-bit analog-to-digital resolution, and an input range of ±562.5 mV. The DAU operates at a supply voltage of 3.7 V, provided by a 700-mAh lithium-polymer (LiPo) battery that supports up to 12 h of continuous operation. Data are transmitted in real time via Bluetooth to an Android monitoring application and simultaneously stored on an onboard SD card for offline analysis.

### 2.4. Signal Preprocessing

**Figure 3:**
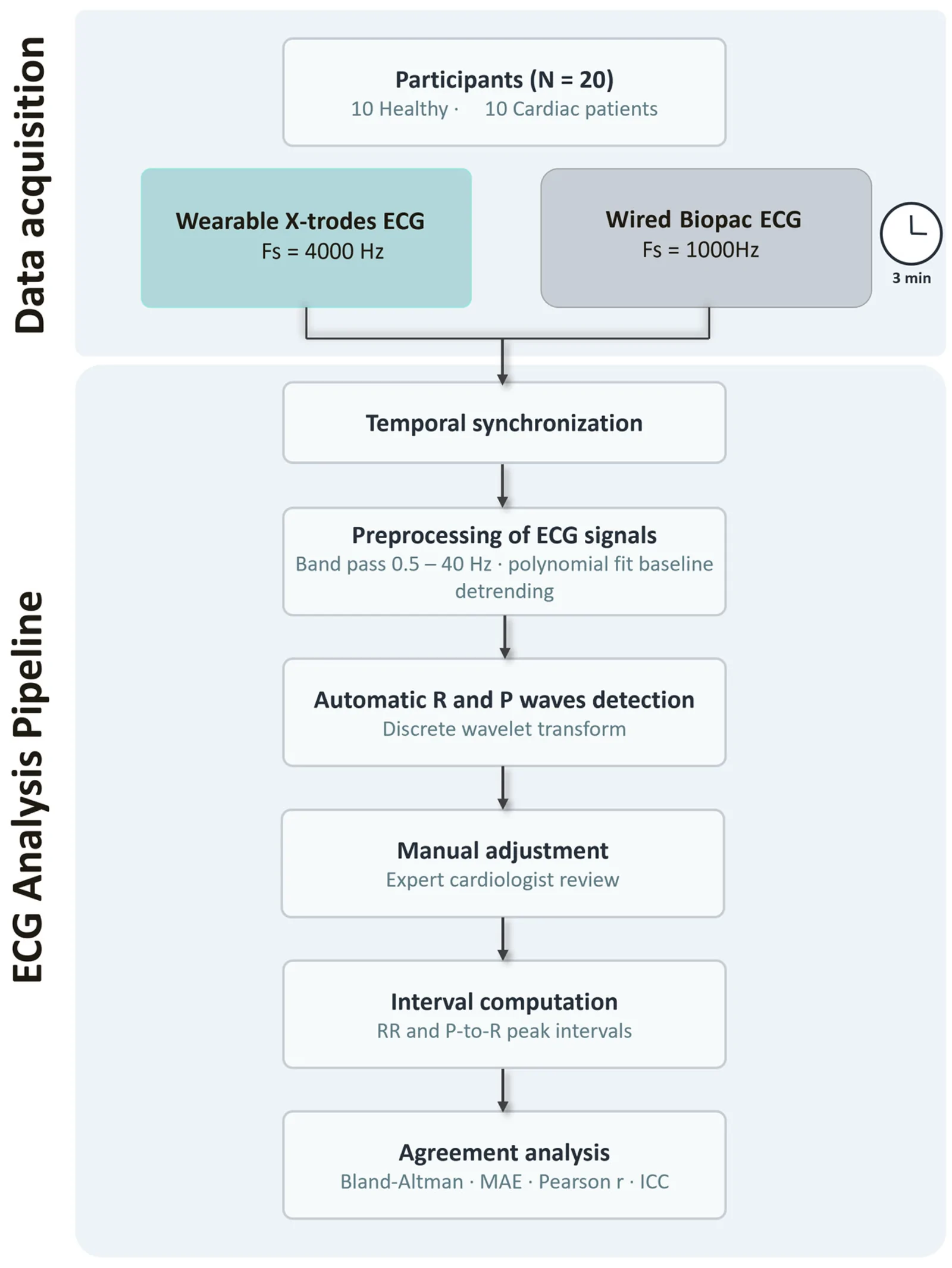
Study workflow. Data acquisition (top): simultaneous 3-min recordings from 20 participants with the wearable X-trodes (Fs = 4000 Hz) and wired Biopac (Fs = 1000 Hz) systems, followed by temporal synchronization. ECG analysis pipeline (bottom): preprocessing, wavelet-based R/P detection, manual adjudication, interval computation, and agreement analysis.

ECG signals from the X-trodes system (4000 Hz) and the Biopac reference system (1000 Hz) were exported in European Data Format (EDF) and processed through a custom Python-based pipeline (Figure 3, ECG analysis pipeline). Initial beat-to-beat temporal alignment ensured the correspondence of cardiac cycles between the systems. The X-trodes raw signals were bandpass-filtered between 0.5 and 40 Hz using a zero-phase, fourth-order Butterworth filter. The lower cutoff of 0.5 Hz was chosen to preserve the low-frequency P-wave components while attenuating the respiration-related baseline drift. The upper cutoff of 40 Hz retained the high-frequency content of the QRS complex while rejecting powerline harmonics and myopotential noise, consistent with established ECG signal-processing standards [27]. A zero-phase filter implementation was used to eliminate group delay, which is critical for accurate interval timing. The filtered X-trodes signals were then decimated from 4000 to 1000 Hz to match the reference sampling rate, and the residual low-frequency drift was removed by detrending with a third-degree polynomial fit. The Biopac reference signal was acquired after filtering by the Biopac system and was therefore analyzed in its hardware-filtered form.

A discrete wavelet transform (DWT)-based delineation approach [34] was implemented using the NeuroKit2 (version 0.2.10) Python (version 3.9) library to detect R- and P-waves [35]. This approach was selected because wavelet-based ECG delineation has been validated for robust P- and R-wave detection across diverse ECG morphologies, including settings in which amplitude-threshold and derivative-based detectors may underperform [34]. Identical algorithmic parameters were applied to both systems, ensuring that any residual detection differences reflected hardware or electrode characteristics rather than computational variation. The processed outputs were exported as CSV files containing time-stamped filtered signals, discrete R- and P-peak annotations, and annotation-status labels.

### 2.5. Manual Adjudication

All automated peak detections underwent standardized manual review using a proprietary synchronized visualization tool (ECG_viewer 3.1), which displayed the wearable and reference ECG signals in a common time window. The reviewers could approve, reject, or delete individual annotations; all modifications were logged with status labels to create a transparent and auditable curation record.

### 2.6. Interval Definition and Cross-Device Matching

RR intervals were defined as the time between consecutive R peaks, consistent with the standards of the Task Force on Heart Rate Variability [26]. P-to-R peak intervals were defined as the time from each detected P-wave peak to the immediately following R peak within the same cardiac cycle. This interval is an algorithmically reproducible timing metric derived from a single bipolar channel and not the conventional diagnostic PR interval measured from P-wave onset to QRS onset on a standard ECG [27]. Intervals were computed independently for each system and matched using a prespecified tolerance window of 100 ms or less. Interval pairs falling outside this window were excluded from the paired agreement analyses and retained for descriptive reporting alone.

### 2.7. Statistical Analysis

Agreement between systems was assessed for RR and P-to-R peak intervals independently in each cohort, on both a per-participant and a pooled within-group basis. The primary analytical method was Bland–Altman analysis [36,37], with 95% limits of agreement (LoA) defined as the mean bias ± 1.96 standard deviations (SDs). This approach was selected over simple correlation because it quantifies both systematic bias and random measurement dispersion. The MAE was computed as a supplementary error metric. The linear association was quantified using the Pearson correlation coefficient (r). Between-group differences in per-subject bias, MAE, and r were assessed using the Mann–Whitney U test because of the small sample sizes and non-normal distribution of the per-subject statistics. The ICC was computed using a two-way mixed-effects, absolute-agreement, single-measure model, following the guidelines in Koo and Li [38], with values exceeding 0.90 interpreted as excellent reliability. All interval values are reported in milliseconds. Pooled within-group analyses combined repeated interval measurements from multiple participants. Because these beat-level observations were clustered within participants, the pooled estimates are presented as descriptive and exploratory summaries rather than as independent participant-level results. The present analysis did not include a fully cluster-adjusted repeated-measures agreement model.

## 3. Results

### 3.1. Participants and Analyzable Data

The cardiac cohort (*n* = 10) had a median age of 77.5 years (range 19–85) and the healthy cohort (*n* = 10) a median age of 41.5 years (range 25–74). The cardiac cohort included patients with structural heart disease comprising ischemic heart disease (*n* = 3), aortic stenosis (*n* = 2), and mitral regurgitation (*n* = 1), as well as conduction abnormalities comprising bundle-branch block (*n* = 2), first-degree atrioventricular block (*n* = 2), atrial fibrillation (*n* = 2), and Wolff–Parkinson–White (WPW) syndrome (*n* = 1). The youngest cardiac participant (aged 19) was the patient with the WPW syndrome, an arrhythmia substrate that can occur in young individuals; this patient was enrolled to evaluate device performance in the setting of accessory pathway-mediated pre-excitation. The baseline characteristics of both cohorts are summarized in Table 1 and Table 2.

One participant from the cardiac cohort was excluded from all analyses because of a technical failure that precluded temporal alignment between systems, leaving nine cardiac patients for the RR analysis. For the P-to-R peak interval analysis, eight healthy volunteers were included, as reliable automated P-wave detection was impeded in two participants by signal noise. Two cardiac patients with atrial fibrillation were also excluded from the P-to-R peak interval analysis because of an absence of discrete P waves, leaving seven cardiac patients for this analysis. In total, 3495 matched RR interval pairs and 2747 matched P-to-R peak interval pairs were available for analysis. These matched pairs represent repeated beat-level measurements clustered within a small number of participants and are not independent participant-level observations.

### 3.2. Representative Waveforms

**Figure 4:**
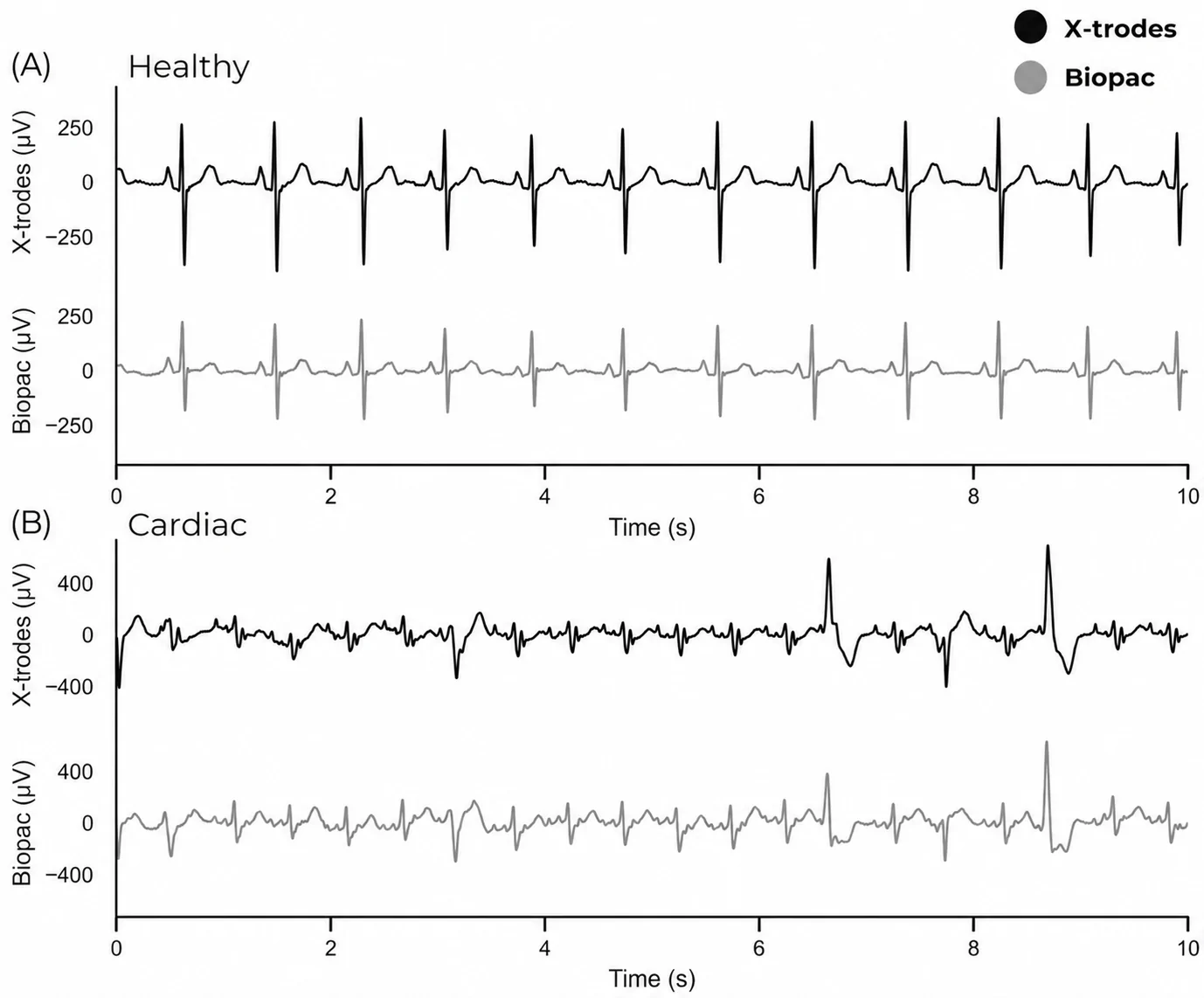
**Representative simultaneous ECG recordings.** The figure shows 10-s traces from the wearable (X-trodes; upper) and wired reference (Biopac; lower) systems in (**A**) a healthy and (**B**) a cardiac patient. In these representative recordings, both systems captured visually concordant beat timing and the major waveform components (P wave, QRS complex, and T wave) in the healthy volunteer, and the more complex morphology associated with cardiac disease in the patient. Amplitude in µV, time in seconds.

Representative 10-s ECG recordings by the wearable (X-trodes) and wired reference (Biopac) systems are shown for a healthy and a cardiac patient in Figure 4. In the healthy subject (Figure 4A), both systems captured visually concordant beat timing and the major waveform components (P wave, QRS complex, and T wave) with a regular rhythm in these representative recordings. In the cardiac patient (Figure 4B), both systems captured the more complex and irregular morphology, including beat-to-beat variability and altered QRS and P-wave configuration that are associated with structural heart disease and conduction abnormality.

### 3.3. RR Interval Agreement

**Figure 5:**
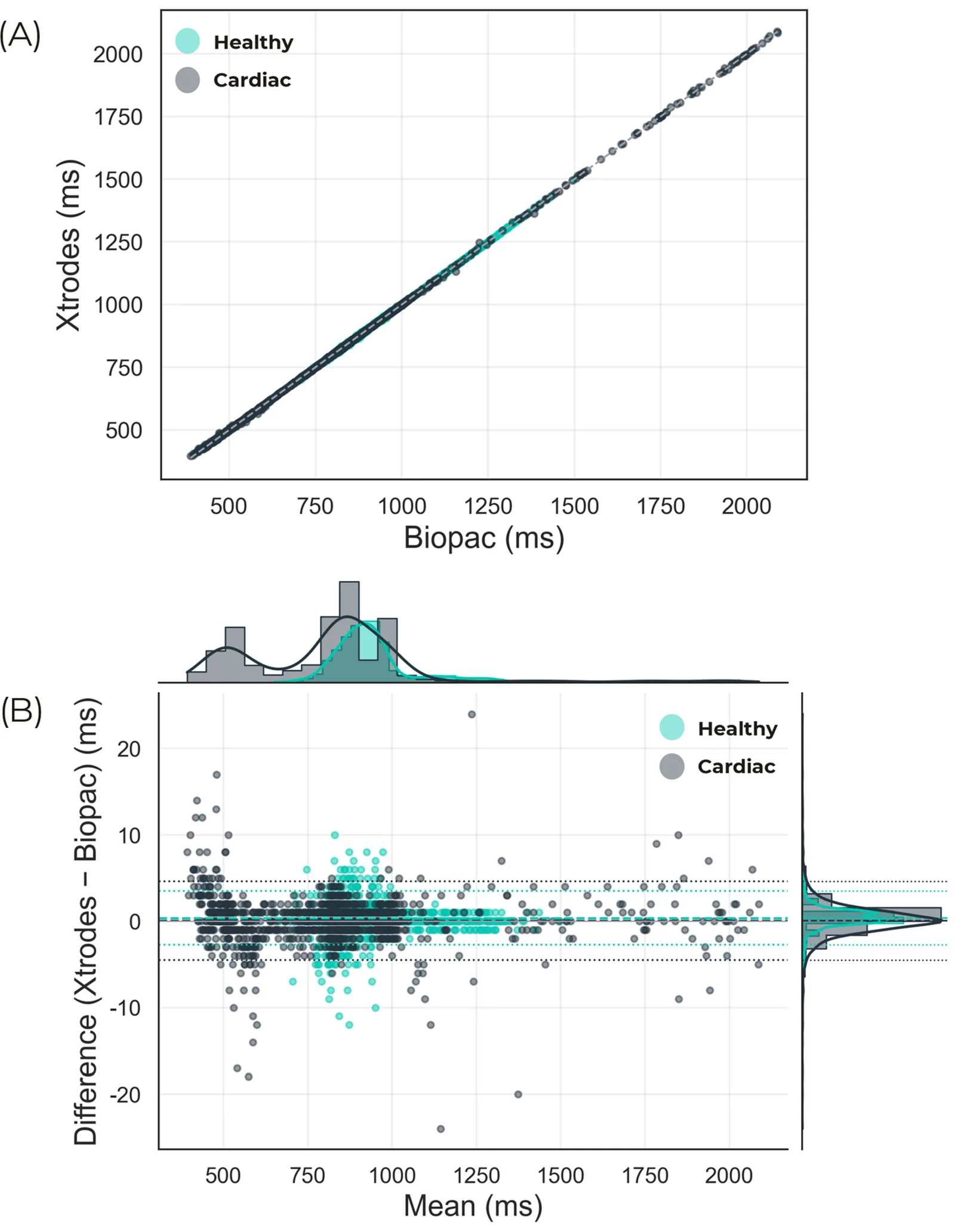
Bland–Altman agreement and correlation analysis for RR intervals. (**A**) Correlation plot. Each point represents a paired RR interval measurement for the healthy (teal) and cardiac (grey) cohorts. The Pearson correlation coefficients exceeded 0.99 for both groups (r = 0.99 for healthy patients; r = 1.0 for cardiac patients). (**B**) Bland–Altman plot showing the difference between systems (*y*-axis) vs. their mean (*x*-axis); horizontal lines indicate mean bias and the 95% limits of agreement. The marginal histograms and density curves show the distribution of measurement differences. No significant between-group differences were observed (*p* > 0.05 for all metrics).

There was excellent agreement between the wearable and reference systems in both cohorts for the RR interval measurements (Table 3; Figure 5). Bland–Altman analysis revealed narrow limits of agreement across the full physiological range of RR values. The Pearson correlation coefficients were r = 0.99 in the healthy cohort and r = 1.0 in the cardiac cohort. The mean bias was 0.4 ± 3.1 ms in the healthy group and 0.1 ± 4.6 ms in the cardiac group, with each cohort’s mean bias smaller than the 1-ms sampling resolution of the reference system, whereas the standard deviations describe the remaining beat-to-beat dispersion. The MAE was 0.97 ms and 1.28 ms, respectively. The ICC exceeded 0.99 in both cohorts, consistent with high inter-system agreement in this sample. No statistically significant between-group differences were observed for bias (*p* = 0.131), MAE (*p* = 0.391), or correlation (*p* = 0.348) in this small sample.

### 3.4. P-to-R Peak Interval Agreement

**Figure 6:**
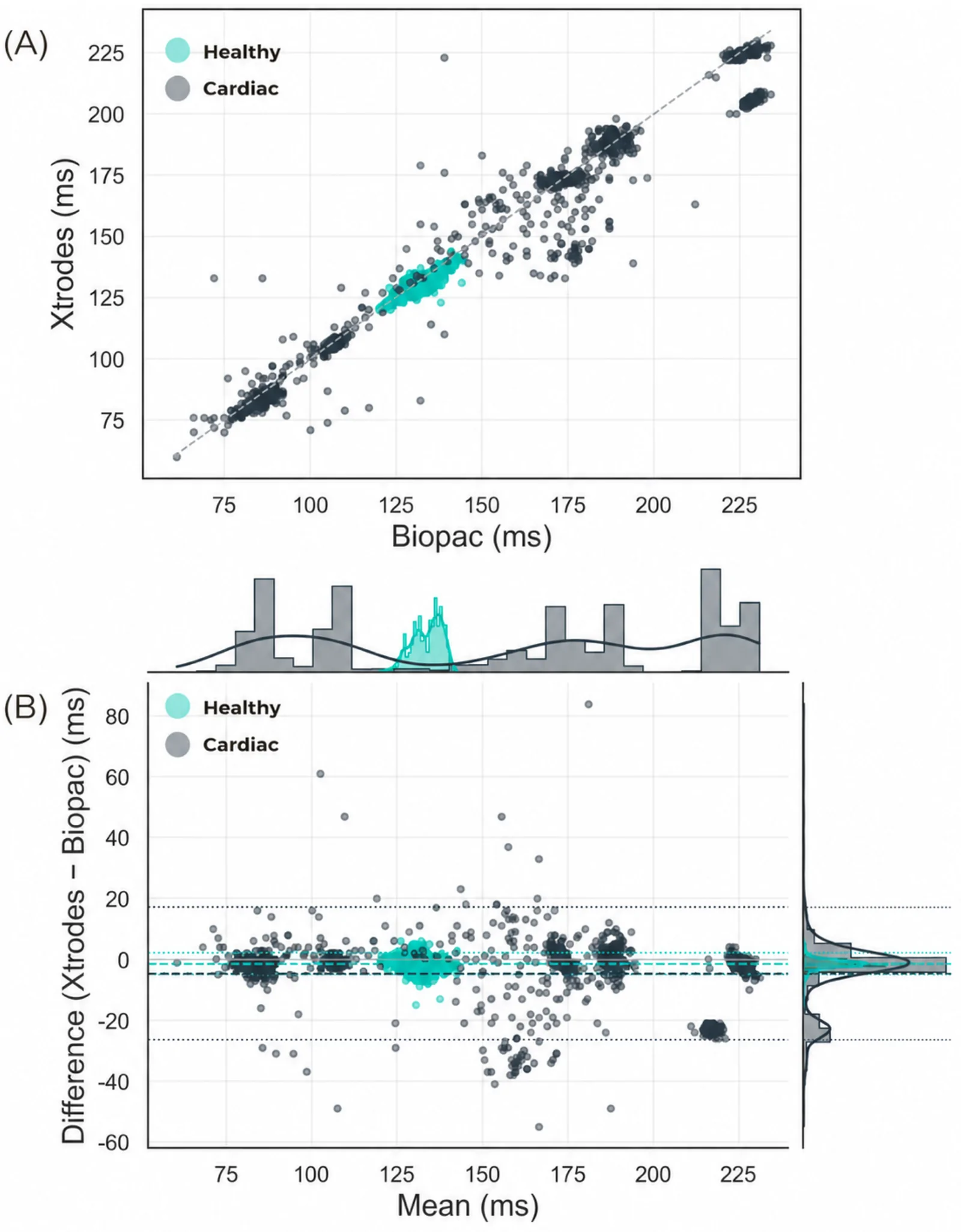
Bland–Altman agreement and correlation analysis for P-to-R peak intervals. (**A**) Correlation plot. Each point represents a paired P-to-R peak interval measurement for the healthy (teal) and cardiac (grey) cohorts. The Pearson coefficients exceeded 0.92 for both groups, indicating a consistent linear relationship. (**B**) Bland–Altman plot showing inter-system differences (*y*-axis) against their mean (*x*-axis), with horizontal lines for the mean bias and the 95% limits of agreement. The marginal histograms and density curves show the distribution of differences. The between-group bias and correlation did not differ significantly (*p* > 0.05); the MAE differed significantly (*p* = 0.040), and the higher MAE in the cardiac cohort was consistent with greater P-wave delineation variability in morphologically complex ECG signals.

The P-to-R peak interval agreement is summarized in Table 4 and illustrated in Figure 6. The Pearson r was 0.92 in the healthy cohort and 0.98 in the cardiac cohort. In the healthy cohort, the mean bias was −1.4 ± 3.5 ms (95% LoA: −8.3 to 5.5 ms) and the MAE was 1.76 ms; the ICC was 0.96. In the cardiac cohort, the mean bias was −4.7 ± 21.8 ms (95% LoA: −47.7 to 38.3 ms) and the MAE was 7.14 ms; the ICC was 0.98. The between-group differences in bias (*p* = 0.779) and correlation (*p* = 0.232) were not statistically significant. The between-group MAE difference was statistically significant (*p* = 0.040), reflecting greater measurement variability in the cardiac cohort, consistent with the known challenges of P-wave delineation in morphologically complex ECG signals.

## 4. Discussion

In this proof-of-concept study, a wearable dry-electrode ECG platform achieved millisecond-level agreement with a wired reference system for RR intervals and strong agreement for P-to-R peak interval measurements when comparing healthy adults to cardiology patients with structural heart disease and conduction abnormalities. This study addresses a less-studied aspect of the wearable ECG literature: published studies have more commonly focused on arrhythmia detection and monitoring feasibility [6,7,8] than on simultaneous, beat-matched interval-agreement analysis against a wired reference, particularly in patients with complex electrophysiological substrates [12].

The RR interval performance was excellent in both cohorts. The observed bias values of 0.4 ± 3.1 ms and 0.1 ± 4.6 ms were each smaller than the 1-ms sampling resolution of the wired reference system, indicating a minimal systematic timing offset under the conditions tested; the reported standard deviations and limits of agreement describe the remaining measurement dispersion. The low MAE values and high ICC estimates support preliminary inter-system agreement for RR interval timing under the tested conditions. The comparable RR performance of the healthy and cardiac cohorts, including patients with bundle-branch block and atrioventricular conduction abnormalities, suggests that RR timing fidelity was preserved within the range of substrates included in this proof-of-concept cohort.

The P-to-R peak interval agreement was strong but showed greater measurement variability in the cardiac cohort. The 95% LoAs were −47.7 to 38.3 ms in the cardiac group versus −8.3 to 5.5 ms in the healthy volunteers, and the MAE was significantly higher (7.14 vs. 1.76 ms; *p* = 0.040). This is consistent with the known difficulty of accurate P-wave timing when P waves are low in amplitude, biphasic, or morphologically aberrant, as can occur in bundle-branch block, atrioventricular block, or WPW syndrome. The between-group difference in mean bias was not statistically significant (*p* = 0.779). The wider limits of agreement in the cardiac group may be related to the challenges of single-lead P-wave delineation in morphologically complex signals; however, the present study was not designed to determine the mechanism underlying this variability.

Future studies should evaluate longer ambulatory and nocturnal recordings under motion, posture, perspiration, and real-world data-completeness conditions, and in clinically relevant populations, before any specific monitoring application can be claimed.

This study was designed with a deliberately narrow scope. It assessed interval-level measurement agreement and did not evaluate arrhythmia classification, diagnostic accuracy, ischemia detection, or morphological ECG analysis, each of which requires dedicated multi-lead validation and prospective clinical outcome studies [26,27]. The two temporal metrics analysed here, the RR interval and the P-to-R peak interval, were prespecified fiducial-point measurements. The RR interval reflects ventricular beat-to-beat timing, whereas the P-to-R peak interval additionally requires reproducible detection of both atrial and ventricular peaks; the latter is a single-lead timing metric rather than the conventional diagnostic PR interval. Conventional PR interval, QRS duration, QT and QTc intervals, ST-segment measurements, waveform morphology, ischemia detection, arrhythmia classification, and comprehensive heart-rate-variability analysis were not added retrospectively, because each requires different or additional methodology, including waveform onset- and offset-adjudication, longer or rhythm-specific recordings, a multi-lead reference, and predefined diagnostic endpoints. The restriction to two prespecified intervals is a deliberate scope boundary that also limits the scientific breadth of the study.

## 5. Limitations

Several limitations should be considered when interpreting the findings. The sample size was small, and the study was designed exclusively for proof-of-concept validation rather than diagnostic evaluation. Twenty participants are insufficient for definitive clinical validation, for population-level generalization, or for adequately powered subgroup analyses; the intraclass correlation values should therefore be interpreted as preliminary estimates requiring confirmation in larger, more heterogeneous cohorts. All recordings were brief (3 min) and were obtained while participants were awake and at rest under controlled daytime conditions. The study therefore evaluated only short-duration recordings and does not establish performance during prolonged, ambulatory, or nocturnal monitoring, sleep, exercise, motion, postural transitions, perspiration, changing skin impedance, multi-day wear, or repeated electrode application. Long-term suitability was neither demonstrated nor excluded and remains unresolved, requiring dedicated future evaluation [30,31,32]. Performance under controlled, static conditions may not reflect performance during ambulatory or prolonged real-world use. The cardiac cohort age range was wide (19 to 85 years), reflecting the heterogeneous referral population, and included one patient aged 19 with WPW syndrome; for the majority of the cardiac participants, the median age was 77.5 years, consistent with an elderly cardiology population.

The P-to-R peak interval analysis was constrained by the exclusion of two cardiac patients (atrial fibrillation and no discrete P waves) and two healthy volunteers (insufficient P-wave detection quality), which reduced the analyzable sample to seven cardiac patients and eight healthy volunteers. The ICC values of 0.96 and 0.98 for P-to-R peak intervals should be interpreted in the context of these small pooled-group estimates. As a proof-of-concept study, a primary limitation lies in the reduced sample size, which warrants a nuanced interpretation of the reported agreement metrics. While the observed intraclass correlation coefficients (ICC of 0.96 and 0.98) demonstrate strong relative consistency between the dry electrodes and the wired reference system, ICC estimates can be highly sensitive to sample size and cohort homogeneity, particularly in smaller or pooled samples.

The high ICC estimates support preliminary inter-system agreement within these recordings, but their precision and generalizability are limited by the small number of participants and the repeated-measures structure of the data. Although several thousand matched interval pairs were available, these represented repeated beat-level measurements clustered within a small participant sample and were not independent participant-level observations; the pooled analyses are therefore descriptive and exploratory, and the absence of a fully cluster-adjusted repeated-measures agreement analysis limits the precision and generalizability of the estimates, including the ICC values.

In addition, because P-to-R peak timing was used as the interval-level metric, the findings should not be interpreted as a validation of conventional diagnostic PR-interval measurement from P-wave onset to QRS onset. The interval-level agreement does not establish equivalence for multi-lead morphological ECG analysis, ischemia localization, or advanced electrophysiological diagnostics, each of which requires dedicated validation [26,27].

This study did not include a comparison with other commercially available wearable ECG devices, which would be needed to contextualize the performance of the X-trodes platform relative to alternatives on the market. Finally, the manual peak-curation step enhanced data accuracy in this validation study but introduced a workflow requirement that must be evaluated in larger, automated deployment settings.

## 6. Conclusions

A wearable dry-electrode ECG platform demonstrated low mean RR bias and strong P-to-R peak interval agreement with a simultaneous wired reference system in healthy volunteers and cardiology patients with structural heart disease and conduction abnormalities. Near-zero RR bias, narrow limits of agreement, and high ICC estimates provide preliminary quantitative evidence of interval-level agreement under controlled, short-duration recording conditions. The P-to-R peak interval agreement was strong, with wider variability in the cardiac cohort, which may relate to the challenges of single-lead P-wave delineation in morphologically complex ECG signals; the present study was not designed to determine the mechanism underlying this variability.

These findings provide a preliminary basis for larger prospective validation of interval-level measurement performance. This platform is not intended to replace multi-lead diagnostic ECG for ischemia assessment, conventional PR-interval diagnosis, or morphological analysis. Larger, multicenter, prospective studies are needed to evaluate performance during longer ambulatory and nocturnal recordings and in higher-risk clinical populations.

## Figures and Tables

**Table 1:** Clinical characteristics of the cardiac cohort (*n* = 10).

Characteristic	Summary Value
**Demographic**	
Age (years), Median [Range]	77.5 [19–85]
Gender (Female/Male)	6/4
BMI (kg/m^2^), Mean ± SD	25.9 ± 4.3
**Structural Heart Disease**	
Ischemic Heart Disease (IHD/MI/CAD)	3
Aortic Stenosis	2
Mitral Regurgitation	1
**Conduction Abnormalities**	
Bundle Branch Blocks (LBBB/RBBBs)	2
Atrioventricular Blocks (1st degree/CAVB)	2
Atrial Fibrillation	2
Wolff–Parkinson–White (WPW)	1

BMI: body mass index; CAD: coronary artery disease; CAVB: complete atrioventricular block; LBBB: left bundle-branch block; RBBB: right bundle-branch block; SD: standard deviation.

**Table 2:** Clinical characteristics of the healthy cohort (*n* = 10).

Characteristic	Summary Value
**Demographic**	
Age (years), Median [Range]	41.5 [25–74]
Gender (Female/Male)	6/4
BMI (kg/m^2^), Mean ± SD	23.4 ± 3.0

BMI: body mass index; SD: standard deviation.

**Table 3:** Statistical analysis of RR interval agreement between systems, by cohort.

RR Interval [ms]	Healthy	Cardiac	MWU *p*-Value
Bias ± SD (ms)	0.4 ± 3.1	0.1 ± 4.6	0.131
95% LoA (ms)	−5.6 to 6.4	−8.9 to 9.1	n/a
Pearson r	0.99	1.0	0.348
ICC	>0.99	>0.99	n/a
MAE (ms)	0.97	1.28	0.391

ICC: intraclass correlation coefficient; LoA: limits of agreement; MAE: mean absolute error; MWU: Mann–Whitney U; SD: standard deviation; n/a: not applicable.

**Table 4:** Statistical analysis of P-to-R peak interval agreement between systems, by cohort.

P-to-R Peak Interval [ms]	Healthy	Cardiac	MWU *p*-Value
Bias ± SD (ms)	−1.4 ± 3.5	−4.7 ± 21.8	0.779
95% LoA (ms)	−8.3 to 5.5	−47.7 to 38.3	n/a
Pearson r	0.92	0.98	0.232
ICC	0.96	0.98	n/a
MAE (ms)	1.76	7.14 *	0.040 *

* Statistically significant between-group difference (*p* < 0.05). ICC: intraclass correlation coefficient; LoA: limits of agreement; MAE: mean absolute error; MWU: Mann–Whitney U; SD: standard deviation; n/a: not applicable.

## Data Availability

De-identified data underlying the analyses may be made available by the corresponding author upon reasonable request, subject to institutional approval and applicable privacy and ethical restrictions.

## References

[1] Zimetbaum P., Goldman A. (2010). Ambulatory arrhythmia monitoring: Choosing the right device. Circulation.

[2] Sandau K.E., Funk M., Auerbach A., Barsness G.W., Blum K., Cvach M., Lampert R., May J.L., McDaniel G.M., Perez M.V. (2017). Update to practice standards for electrocardiographic monitoring in hospital settings: A scientific statement from the American Heart Association. Circulation.

[3] Hindricks G., Potpara T., Dagres N., Arbelo E., Bax J.J., Blomström-Lundqvist C., Boriani G., Castella M., Dan G.-A., E Dilaveris P. (2021). 2020 ESC Guidelines for the diagnosis and management of atrial fibrillation. Eur. Heart J..

[4] January C.T., Wann L.S., Calkins H., Chen L.Y., Cigarroa J.E., Cleveland J.C., Ellinor P.T., Ezekowitz M.D., Field M.E., Furie K.L. (2019). 2019 AHA/ACC/HRS focused update of the 2014 AHA/ACC/HRS guideline for the management of patients with atrial fibrillation. Circulation.

[5] Kusumoto F.M., Schoenfeld M.H., Barrett C., Edgerton J.R., Ellenbogen K.A., Gold M.R., Goldschlager N.F., Hamilton R.M., Joglar J.A., Kim R.J. (2019). 2018 ACC/AHA/HRS guideline on the evaluation and management of patients with bradycardia and cardiac conduction delay. Circulation.

[6] Turakhia M.P., Hoang D.D., Zimetbaum P., Miller J.D., Froelicher V.F., Kumar U.N., Xu X., Yang F., Heidenreich P.A. (2013). Diagnostic utility of a novel leadless arrhythmia monitoring device. Am. J. Cardiol..

[7] Barrett P.M., Komatireddy R., Haaser S., Topol S., Sheard J., Encinas J., Fought A.J., Topol E.J. (2014). Comparison of 24-hour Holter monitoring with 14-day novel adhesive patch electrocardiographic monitoring. Am. J. Med..

[8] Steinhubl S.R., Waalen J., Edwards A.M., Ariniello L.M., Mehta R.R., Ebner G.S., Carter C., Baca-Motes K., Felicione E., Sarich T. (2018). Effect of a home-based wearable continuous ECG monitoring patch on detection of undiagnosed atrial fibrillation: The mSToPS randomized clinical trial. JAMA.

[9] Kim D.H., Lu N., Ma R., Kim Y.-S., Kim R.-H., Wang S., Wu J., Won S.M., Tao H., Islam A. (2011). Epidermal electronics. Science.

[10] Chi Y.M., Jung T.P., Cauwenberghs G. (2010). Dry-contact and noncontact biopotential electrodes: Methodological review. IEEE Rev. Biomed. Eng..

[11] Meziane N., Webster J.G., Attari M., Nimunkar A.J. (2013). Dry electrodes for electrocardiography. Physiol. Meas..

[12] Hughes A., Shandhi M.M.H., Master H., Dunn J., Brittain E. (2023). Wearable devices in cardiovascular medicine. Circ. Res..

[13] Kumar G., Duggal B., Singh J.P., Shrivastava Y. (2025). Efficacy of various dry electrode-based ECG sensors: A review. J. Biomed. Mater. Res. A.

[14] Fu Y., Zhao J., Dong Y., Wang X. (2020). Dry electrodes for human bioelectrical signal monitoring. Sensors.

[15] Joutsen A., Cömert A., Kaappa E., Vanhatalo K., Riistama J., Vehkaoja A., Eskola H. (2024). ECG signal quality in intermittent long-term dry electrode recordings with controlled motion artifacts. Sci. Rep..

[16] Nigusse A.B., Mengistie D.A., Malengier B., Tseghai G.B., Van Langenhove L. (2021). Wearable smart textiles for long-term electrocardiography monitoring—A review. Sensors.

[17] Machino T., Aonuma K., Komatsu Y., Yamasaki H., Igarashi M., Nogami A., Ieda M. (2022). Dry textile electrode for ambulatory monitoring after catheter ablation of atrial fibrillation. F1000Research.

[18] Arquilla K., Webb A.K., Anderson A.P. (2020). Textile electrocardiogram (ECG) electrodes for wearable health monitoring. Sensors.

[19] Soroudi A., Hernández N., Berglin L., Nierstrasz V. (2019). Electrode placement in electrocardiography smart garments: A review. J. Electrocardiol..

[20] Singh O.P., Bocchino A., Guillerm T., O’Mahony C. (2022). Design, fabrication and performance assessment of flexible, microneedle-based electrodes for ECG signal monitoring. Annu. Int. Conf. IEEE Eng. Med. Biol. Soc..

[21] Zhang H., Pei W., Chen Y., Guo X., Wu X., Yang X., Chen H. (2016). A motion interference-insensitive flexible dry electrode. IEEE Trans. Biomed. Eng..

[22] Majumder S., Chen L., Marinov O., Chen C.H., Mondal T., Deen M.J. (2018). Noncontact wearable wireless ECG systems for long-term monitoring. IEEE Rev. Biomed. Eng..

[23] Bujnowski A., Osiński K., Przystup P., Wtorek J. (2022). Non-contact monitoring of ECG in the home environment—Selecting optimal electrode configuration. Sensors.

[24] Oz S., Dagay A., Katzav S., Wasserman D., Tauman R., Gerston A., Duncan I., Hanein Y., Mirelman A. (2023). Monitoring sleep stages with a soft electrode array: Comparison against video-PSG and home-based detection of REM sleep without atonia. J. Sleep Res..

[25] Shustak S., Inzelberg L., Steinberg S., Rand D., Pur M.D., Hillel I., Katzav S., Fahoum F., De Vos M., Mirelman A. (2019). Home monitoring of sleep with a temporary-tattoo EEG, EOG and EMG electrode array: A feasibility study. J. Neural. Eng..

[26] Task Force of the European Society of Cardiology and the North American Society of Pacing and Electrophysiology (1996). Heart rate variability: Standards of measurement, physiological interpretation and clinical use. Circulation.

[27] Kligfield P., Gettes L.S., Bailey J.J., Childers R., Deal B.J., Hancock E.W., Van Herpen G., Kors J.A., Macfarlane P., Mirvis D.M. (2007). Recommendations for the standardization and interpretation of the electrocardiogram: Part I: The electrocardiogram and its technology. Circulation.

[28] Dahiya E.S., Kalra A.M., Lowe A., Anand G. (2024). Wearable technology for monitoring electrocardiograms (ECGs) in adults: A scoping review. Sensors.

[29] Liu T., Hu X., Wang D., Shi W., Xing D., You C., Xu T., Tian M., Qu L., Zhang X. (2025). Flexible dry electrodes for electrocardiographic monitoring: Working principles, design strategies, and recent advances. iScience.

[30] Ravichandran V., Ciesielska-Wrobel I., Rumon M.A.A., Solanki D., Mankodiya K. (2023). Characterizing the impedance properties of dry e-textile electrodes based on contact force and perspiration. Biosensors.

[31] Zhao Y., Zhang S., Yu T., Zhang Y., Ye G., Cui H., He C., Jiang W., Zhai Y., Lu C. (2021). Ultra-conformal skin electrodes with synergistically enhanced conductivity for long-time and low-motion artifact epidermal electrophysiology. Nat. Commun..

[32] Jurado I.C., Lorato I., Morales J., Fruytier L., Stuart S., Panditha P., Janssen D.M., Rossetti N., Uzunbajakava N., Serban I.B. (2023). Signal quality analysis for long-term ECG monitoring using a health patch in cardiac patients. Sensors.

[33] U.S. Food and Drug Administration (2024). 510(k) Premarket Notification K232210: X-Trodes System M.

[34] Martinez J.P., Almeida R., Olmos S., Rocha A.P., Laguna P. (2004). A wavelet-based ECG delineator: Evaluation on standard databases. IEEE Trans. Biomed. Eng..

[35] Makowski D., Pham T., Lau Z.J., Brammer J.C., Lespinasse F., Pham H., Schölzel C., Chen S.H.A. (2021). NeuroKit2: A Python toolbox for neurophysiological signal processing. Behav. Res. Methods.

[36] Bland J.M., Altman D.G. (1986). Statistical methods for assessing agreement between two methods of clinical measurement. Lancet.

[37] Bland J.M., Altman D.G. (1995). Comparing methods of measurement: Why plotting difference against standard method is misleading. Lancet.

[38] Koo T.K., Li M.Y. (2016). A guideline of selecting and reporting intraclass correlation coefficients for reliability research. J. Chiropr. Med..

